# Community Drivers of *Leishmania* Infection, Transmission and Control in Baringo County, Kenya: Implications for Integrated One Health Intervention Strategies

**DOI:** 10.3390/tropicalmed11070197

**Published:** 2026-07-14

**Authors:** Hellen Njeri Maingi, Maingi Ndichu, James Ng’ang’a Chege, Davis Njuguna Karanja, Derrick Noah Sentamu, Bruno Enagnon Lokonon, Damaris Matoke-Muhia, Helena Ngowi, Bassirou Bonfoh

**Affiliations:** 1 Department of Veterinary Pathology, Microbiology and Parasitology, University of Nairobi, Nairobi P.O. Box 29053-00625, Kenya; nmaingi@uonbi.ac.ke (M.N.); jchege@uonbi.ac.ke (J.N.C.); dnkaranja@uonbi.ac.ke (D.N.K.); 2 Department of Public Health Pharmacology and Toxicology, University of Nairobi, Nairobi P.O. Box 29053-00625, Kenya; sentsderrick@gmail.com; 3 Laboratoire de Biomathématiques et d’Estimations Forestières, Faculté of Agronomiques Sciences, Université d’Abomey-Calavi, Abomey-Calavi P.O. Box 04 BP 1525, Benin; brunolokonon@gmail.com; 4 Centre for Biotechnology Research and Development, Kenya Medical Research Institute, Nairobi P.O. Box 54840-00200, Kenya; dmatoke@kemri.go.ke; 5 Department of Veterinary Medicine and Public Health, Sokoine University of Agriculture, Morogoro P.O. Box 3000, Tanzania; helenangowi@gmail.com; 6 Centre Suisse de Recherches Scientifiques en Côte d’Ivoire (CSRS), Abidjan P.O. Box 01 BP 1303, Côte d’Ivoire; bassirou.bonfoh@csrs.ci

**Keywords:** leishmaniasis, KAP survey, neglected tropical disease, vector-borne disease sandflies, reservoir host, one health, Kenya

## Abstract

Leishmaniasis is an endemic zoonotic disease caused by the protozoan parasite *Leishmania* and transmitted by infected female phlebotomine sandflies. The World Health Organization (WHO) classifies it among the 20 neglected tropical diseases (NTDs), ranking eighth globally. The disease predominantly affects resource-limited populations in tropical regions. Kenya bears a significant global burden of leishmaniasis, with Baringo County identified as a major endemic area. A cross-sectional survey on knowledge, attitudes, and practices (KAP) was conducted from July 2025 to January 2026 among 135 residents of Baringo South Sub-County using a structured questionnaire. The study evaluated sociodemographic characteristics, disease awareness, perceptions, preventive practices, and treatment-seeking behaviors. Additionally, 12 healthcare professionals were interviewed regarding their diagnostic and treatment capacities. Associations between variables were analyzed using Chi-square tests and multivariate logistic regression. Overall awareness of leishmaniasis was high (94.8%), with KAP scores of 86.7%, 76%, and 77.7%, respectively. Despite this, critical knowledge gaps persisted regarding transmission dynamics, the role of dogs as reservoir hosts, and preventive measures. Significant associations were found for awareness of local cases, recognition of clinical signs, knowledge of vector activity, community control measures, and attitudes toward treatment preferences. Age and education level predicted KAP outcomes. Healthcare practitioners demonstrated technical capacity to diagnose and treat human *Leishmania* infections, whereas veterinarians lacked awareness of *Leishmania* infection in dogs, including its diagnosis and treatment. Integration of human, animal, and vector surveillance within a One Health framework is recommended to enhance disease monitoring and control in Baringo County.

## 1. Introduction

Leishmaniasis is a vector-borne zoonotic parasitic disease transmitted by the bites of infected female phlebotomine sandflies. It is considered an emerging endemic and is classified among neglected tropical diseases (NTDs), representing a significant public health concern in many tropical and subtropical regions. The disease is caused by more than 20 species of the genus *Leishmania*, including *L. donovani* and *L. infantum* for visceral leishmaniasis (VL), as well as *L. major*, *L. tropica*, and *L. aethiopica* for cutaneous leishmaniasis (CL). Leishmaniasis manifests in several clinical forms, such as visceral leishmaniasis (VL), cutaneous leishmaniasis (CL), and mucocutaneous leishmaniasis (MCL) [1].

Canines are the primary reservoir hosts, but other reservoirs include rodents, marsupials, and foxes for the zoonotic visceral leishmaniasis caused by *L. infantum.* Humans on the other hand are the reservoir host for the anthroponotic visceral leishmaniasis (AVL), caused by *L. donovani*. However, community awareness of canines as reservoirs is often limited. This lack of awareness, combined with the close relationship between humans and domestic animals in rural areas, increases the risk of vector-borne transmission [2].

The parasite is transmitted through complex anthroponotic or zoonotic cycles involving sandfly vectors, mammalian hosts, and environmental conditions that support vector survival. Transmission occurs when an infected female phlebotomine sandfly injects metacyclic promastigotes into the host’s skin during a blood meal. Both biological and environmental factors influence this host–vector–parasite interaction, determining the persistence and spread of the disease [3,4].

According to the World Health Organization (WHO), leishmaniasis has been listed at eighth position among the 20 priority NTDs in humans targeted for control and elimination by 2030 [5,6]. The disease affects marginalized, impoverished, and malnourished populations in arid and semi-arid rural areas with poor access to healthcare, and who live in poorly constructed houses. It is also endemic among communities that keep animals and lack knowledge of disease, vector behavior, and protective measures [7,8,9,10].

The disease has a global distribution affecting millions of neglected communities and placing more than one billion individuals at risk of infection. Leishmaniasis is endemic in 99 countries worldwide across Asia, Africa, the Middle East, Central and South America, and the Mediterranean. There is an estimated overall 700 to 1 million new cases for both VL and CL worldwide annually, according to the WHO. VL accounts for 50,000–90,000 new cases, while CL account for 600,000–1 million new cases per year [11,12]. Globally, there are estimates of about 12 million existing cases, about 40,000–50,000 mortalities annually, and more than 350 million people at risk of contracting the disease [11,13,14]. 

East Africa is among the region’s most severely affected by both VL and CL, with countries such as Ethiopia, Sudan, Somalia, Eritrea, and Kenya reporting high disease burdens. In Kenya, the disease is endemic in several counties, including Baringo, Turkana, Marsabit, Wajir, Isiolo, Kitui, West Pokot, and Garissa. Baringo County, situated in the semi-arid Rift Valley, offers ecological conditions conducive to sandfly resting and breeding, owing to the presence of termite mounds, vegetation, and traditional housing structures. These factors increase human exposure to sandfly vectors. Additionally, livestock keeping and dog ownership are prevalent in the county, providing reservoirs of animals and potentially contributing to the persistence of infections [12,15,16].The effectiveness of leishmaniasis control depends on the KAP of affected communities, as well as on identifying existing gaps, which are crucial for designing and implementing effective interventions. Sufficient capacity among healthcare practitioners for early detection, diagnosis, and treatment of the disease is also vital. This study aimed to conduct a comprehensive assessment of the socio-epidemiological, behavioral, and institutional factors influencing visceral leishmaniasis in Baringo County, Kenya. The study identified critical gaps in community knowledge regarding zoonotic transmission, particularly the underrecognized role of domestic dogs, and evaluating operational constraints within the local healthcare system, thereby supporting the need for an integrated One Health surveillance and control framework.

## 2. Materials and Methods

### 2.1. Description of the Study Area

The study was conducted in Baringo South Sub-County in Baringo County, Kenya. The study area was purposely selected because of its accessibility, adequate security, and characteristic environmental conditions favorable for sandfly breeding, as well as pastoral activities and dog ownership. There have also been previous reports of the area being the main endemic foci for VL and CL transmission to neighboring counties [17]. However, the population of dogs in the study areas was not well documented in the Kenya National Bureau of Statistics 2019 census. Still, preliminary findings on *Leishmania* status have been reported, although not published.

Baringo County covers approximately 11,075 km^2^ with an estimated human population of 555,561 [18]. The region lies about 250 km northwest of Nairobi at an altitude of approximately 1067 m above sea level. The county experiences semi-arid climatic conditions, with mean annual temperatures of 32.8 ± 1.6 °C and average annual rainfall of approximately 512 mm, occurring in two rainy seasons (March–August and November–December).

The environmental conditions in the area support sandfly breeding, with features such as termite mounds, animal burrows, rock crevices, tree holes, and dense vegetation. Traditional housing structures with mud-plastered walls and thatched roofs also provide suitable resting sites for sandflies, thereby increasing the risk of human exposure [19].

The main economic activities are predominantly mixed farming, livestock keeping, pastoralism, and trading, which may increase exposure to sandflies and potential reservoir hosts. Healthcare infrastructure is limited, as most residents belong to pastoral communities living in remote rural areas. This situation imposes financial burdens related to medication and transportation to healthcare facilities by perpetuating cycles of poverty. Additionally, some residents have limited knowledge of disease transmission and prevention methods, such as the use of insecticide-treated nets, and of cultural practices like sleeping outdoors, which further elevate the risk [20].

**Figure 1:**
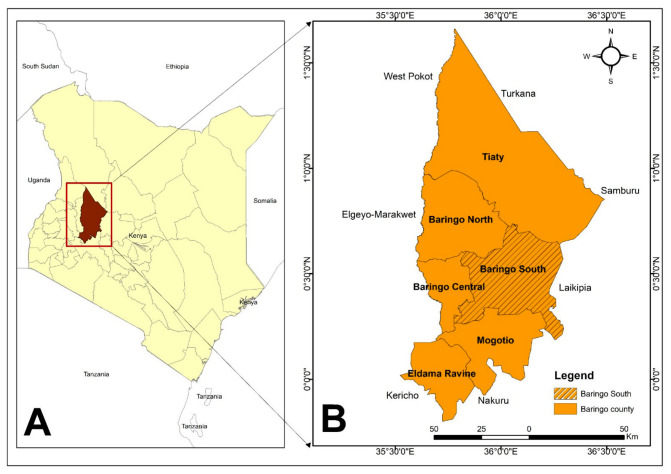
Map of Kenya (**A**) showing the location of Baringo County and inset (**B**) map of Baringo County, showing its sub-counties and the neighboring counties.

Baringo County shares boundaries with the following counties: Turkana and West Pokot to the north, Samburu and Laikipia to the east, Nakuru and Kericho to the south, Uasin Gishu to the southeast, and Elgeyo Marakwet to the west. Administratively, the county is divided into seven sub-counties: Baringo South, Baringo Central, Baringo North, Mogotio, Eldama Ravine and Tiaty (Figure 1).

### 2.2. Study Population

The study population consisted of residents of Baringo South Sub-County and healthcare workers who had worked within the study area over the last year.

### 2.3. Study Design

#### 2.3.1. Study Design and Location Selection

This study was a cross-sectional questionnaire survey conducted between July 2025 and January 2026, adopting a One Health approach to integrate human, animal, and environmental transmission factors. The study selected four locations in Baringo South County based on their specific epidemiological and research profiles. These included the Marigat location, selected as a documented high-burden hotspot, where prevalence is linked to environmental features such as termite hills [20]. Kimalel was selected for its historical role as a primary visceral leishmaniasis treatment and research hub, hosting major clinical trials and vaccine projects (Prev_PKDL) supported by DNDi and KEMRI [21,22] Sandai and Kapkuikui locations were included due to established high seroprevalence in nearby villages like Loboi and Parkarin and their representation of endemic lowland ecologies [23]. The selection also accounted for security and accessibility, as tribal clashes and cattle rustling in Baringo often necessitate purposive site selection to ensure researcher safety while reaching hard-to-access populations [24].

#### 2.3.2. Sampling Strategy and Household Selection

The study employed a multi-stage random sampling approach, identifying specific sub-locations and villages within each location. Household lists were compiled from village elders, community health workers, and the KNBS, which served as a sampling frame. A systematic sampling that involved the selection of every third household was used to ensure representativeness in sparsely populated pastoralist settlements [25,26]. The household heads were prioritized as they are the primary decision-makers regarding the socio-economic and pastoral activities that directly influence the risk of leishmaniasis [20,27].

#### 2.3.3. Assessment of KAP and Behavioral Factors

The survey measured the “Knowledge, Attitudes, and Practices” of the community, specifically focusing on knowledge gaps in community awareness of infection sources, transmission mechanisms (sand fly vector), symptoms, prevention, and their sources of information about leishmaniasis, e.g., healthcare providers, community meetings, or media. It also measured community perceptions of cultural beliefs, traditional explanations, and the stigma and misconceptions surrounding treatment-seeking behavior, as well as their behavioral risks factors and practices, such as sleeping outside or near animals, use of insecticide-treated bed nets, environmental sanitation, and animal husbandry practices, including migrations that increase the risk of vector exposure.

#### 2.3.4. Key Informant Interviews

This study also assessed the county’s health capacity using purposive key informant interviews and cross-sectional sampling of experts from human and veterinary health sectors. Healthcare professionals included doctors, clinicians, nurses, public health officers, and health record and laboratory technicians from endemic sub-counties, each with at least one year of experience managing visceral or cutaneous leishmaniasis at established centers [21,24]. Veterinary officers included registered veterinarians and animal health assistants from the Baringo County Department of Agriculture, Livestock, and Fisheries, responsible for zoonotic disease surveillance in pastoralist zones [19,27]. The aim was to gather data from both clinical and veterinary perspectives to inform the One Health Integration strategy and provide evidence-based recommendations for multidisciplinary surveillance and targeted control of persistent leishmaniasis outbreaks in Kenya.

### 2.4. Sample Size Determination and Justification

The sample size was calculated using Cochran’s formula for a cross-sectional survey at a 95% confidence level, based on a 21% prevalence estimate from similar East African pastoral communities, to ensure sufficient statistical power for identifying socio-environmental risk factors in the endemic Baringo South Sub-County [28]. A 7% margin of error was selected instead of the standard 5% to accommodate logistical challenges, low population density, and the mobile nature of pastoralist populations in Baringo South Sub-County [19,29]. A total of 135 participants were included, which was considered adequate to capture key socio-environmental risk factors while allowing for potential non-response. The small, targeted sample size enabled high-quality face-to-face data collection, reducing misinterpretation and nonresponse that are common in surveys involving mobile and hard-to-reach pastoralist populations in areas with varying literacy levels. Additionally, the homogeneity of knowledge and practices stems from shared cultural traditions and long-term disease exposure in this endemic community in Baringo County [25,30].

Additionally, 10 healthcare professionals and 2 veterinary professionals were included for key informant interviews to achieve saturation and expert representation. A total of 12 professionals was considered sufficient because the healthcare workers represented a considerable proportion of the county’s clinical staff directly involved in leishmaniasis management. At the same time, the 2 veterinarians provided adequate veterinary oversight. In addition, there were constraints of professional staffing in these remote, semi-arid regions [30].

### 2.5. Data Collection

A pretested and validated semi-structured questionnaire, originally written in English and translated into Kalenjin by a trained research assistant, was administered using Open Data Kit (ODK). The questionnaire collected data on respondents’ sociodemographic characteristics and their awareness, beliefs, and practices regarding leishmaniasis. The study further aimed to identify community control measures, including vegetation clearance and destruction of termite hills near homesteads. Additionally, a trained research assistant used a checklist to assess environmental and household conditions, including proximity to vector breeding sites and the nature of housing structures.

Key informant interviews were conducted with medical health workers to gather information on the number of leishmaniasis cases managed within one year, diagnostic methods employed, available treatment options, and sources of anti-*Leishmania* drugs for the facility.

### 2.6. Inclusion Criteria

#### 2.6.1. Household Respondents

The study included male and female respondents aged 18 years or older who were either household heads or alternative adult household members available at the time of the survey. Eligibility required participants to have resided in the study area for at least one year to ensure that the findings reflect sustained exposure to the local environment and community-specific behavioral risks.

#### 2.6.2. Key Informants

Eligible healthcare professionals must be registered and currently employed at one of the purposively selected facilities in the endemic sub-counties, such as Kimalel Health Center, Marigat Sub-County Hospital, or Chemolingot Sub-County Hospital. In addition, they must have at least 1 year of experience in leishmaniasis detection, diagnostic testing, and treatment. Veterinarians and Animal Health Officers must be registered and serving under the Baringo County Department of Agriculture, Livestock, and Fisheries, with specific oversight of the Baringo South or Tiaty East/West sub-counties. Relevant experience in surveillance or management of zoonotic diseases within pastoralist communities is also required.

### 2.7. Participant Consent

Informed consent was sought from the study participants after the study protocol was explained to them. The participants were also assured of the confidentiality of any details shared with the researcher, as well as of the study’s findings and their benefits to the control of disease in the area. The participants who voluntarily agreed to take part in the study signed a written consent form indicating their willingness to participate.

### 2.8. KAP Scoring System

Participants’ knowledge, attitude, and practice were assessed using structured scoring criteria adapted from previous KAP studies [31]. Awareness was measured using 12 questions, with a maximum of 20 points, categorized as follows: 0–5 (poor), 6–10 (basic), 11–15 (moderate), and 16–20 (good). Attitudes were assessed using five Likert-scale statements, with a maximum score of 6 points; scores of 0–2 indicated a negative attitude, 3–4 a moderate attitude, and 5–6 a positive attitude. Preventive practices were measured with six behavioral questions, with a maximum score of 12 points, scored as 0–4 (poor), 5–8 (fair), and 9–12 (good).

### 2.9. Data Management and Statistical Analysis

Data was collected electronically by trained data collectors using Open Data Kit (ODK) (V2026.2.2) and stored in a secure cloud database. The data collected from each respondent after each session was checked to ensure the session was completed before starting the next interview. Statistical analysis was done using statistical software R 4.4.1 [32]. The analysis included descriptive statistics of frequencies, proportions, medians, means, ranges and percentages and inferential statistics were used to explore the associations. Univariate analysis was done using the Arsenal package in R [33], with the criterion of ever having been diagnosed with leishmaniasis as the outcome variable. Predictor variables that returned with statistical *p* values ≤ 0.1 were proceeded for further analysis to a binomial logistic regression model and analyzed with the DescTools package [34]. *p* values for statistical significance were set at *p* ≤ 0.05, and odds ratios of associated variables were reported. The same procedure described above was followed when examining associations among sociodemographic characteristics. However, if the outcome variable had more than 3 categories/levels, the significant univariate variables were subjected to multinomial logistic regression using the package VGAM [35].

## 3. Results

### 3.1. Associations Between Sociodemographic Characteristics and Knowledge, Attitude and Practices of the Respondents

A total of 135 respondents took part in the study, and all of them were from the Kalenjin ethnic group, which is predominant in the county. Most respondents (97%) had lived in the study area for more than 1 year, indicating a relatively stable population with long-term exposure to the local environment and potential disease risk factors. There were more men (55.6%) than women, and the mean age was 42.6 years (SD = 14.6; range 18–83). A substantial number (43.7%) of the respondents had reached at least a primary school level of education. Most respondents were married (74.1%), while 14.1% were single and 11.1% were widowed. The predominant economic activity was mixed farming (56.3%), followed by casual labor (18.5%) and trading (15.6%). No statistically significant associations were seen between sociodemographic characteristics and earlier diagnosis of leishmaniasis (*p* > 0.05) (Table 1).

The multivariate analysis found age and education level as the only statistically significant predictors of the respondents’ KAP. Younger respondents were more likely to report “poor” to “fair” attitudes toward the disease than older residents (OR = 1.007–1.037). Respondents with any level of basic education were significantly less likely to hold poor or fair attitudes than those with no formal education, with notably low odds ratios among those with secondary and tertiary education (Table 1).

### 3.2. Knowledge and Awareness of Leishmaniasis

The study found high awareness of leishmaniasis (94.8%), with 86.7% of respondents demonstrating moderate to good knowledge. Information was primarily obtained through community members (42.2%), followed by healthcare workers (29.6%) and broadcast media (15.6%). Most participants recognized common clinical signs and were aware of local disease cases, indicating strong disease recognition. However, important knowledge gaps remained regarding transmission and prevention. Only 31.9% correctly identified sand flies as the vector, while misconceptions about vector sources and activity periods were common. Awareness of preventive measures was moderate, with less than half of respondents recognizing personal or community-level prevention strategies. The greatest knowledge gap was the disease’s zoonotic nature, as 99.3% of respondents were unaware that dogs are important reservoir hosts (Table 2).

### 3.3. Community Attitudes and Perceptions Toward Leishmaniasis

The study found that 76% of respondents exhibited a generally positive attitude toward leishmaniasis management. A majority (82.2%) indicated a positive intent to seek medical treatment upon experiencing symptoms, and an identical percentage recognized leishmaniasis as a significant public health burden. Most participants (68.9%) preferred modern hospital-based medical treatment, whereas 13.3% continued to rely on traditional herbal remedies. Despite the preference for modern medicine, 68.9% of respondents perceived treatment as costly. A notable disconnect emerged between individual- and community-level prevention efforts: more than half of respondents (60%) reported a negative attitude toward personal protective measures, such as using repellents or specific clothing, highlighting substantial behavioral barriers to individual-level vector control. In contrast, there was a statistically significant willingness to participate in community-based activities. More than half of respondents expressed interest in collective interventions, specifically vegetation clearing and the destruction of termite mounds, which serve as breeding sites for sand flies in the region (Table 3).

### 3.4. Preventive Practices and Behavioral Risk

The study population demonstrated favorable preventive practice scores (77.7%). A substantial proportion of respondents (96.3%) reported sleeping under mosquito nets, which may provide indirect protection. Environmental hygiene practices were also positive, with 95.6% of participants disposing of waste by burning or burying. However, 29.6% of respondents reported using traditional treatments, including medicinal herbs and tree bark, which may delay clinical diagnosis.

Significant behavioral risks were identified within households: 81.5% of respondents reported owning dogs and 24.4% reported sleeping near their dogs at night. Additionally, 93.3% of participants rear domestic animals, including cattle, sheep, and goats. The study also observed elevated levels of population mobility, with 56.3% of respondents indicating periodic migration due to pastoralism, farming, trade, or tribal conflicts (Table 4).

### 3.5. Logistic Regression Analysis of the Predictors of KAP

Variables with *p* ≤ 0.1 in the univariate analysis were included in the final multivariable regression model to identify independent predictors of infection risk. Logistic regression analysis shows that the prominent predictor identified was unawareness of the infection source, where respondents who were unaware of the source of infection were 7.77 times more likely to have been diagnosed with leishmaniasis (OR = 7.77; 95% CI: 1.42–52.87, *p* = 0.018) as compared to those who correctly identified sandflies as the source. Significant odds were also registered for awareness of reported cases, number of cases, sources of infection such as termite hills and sandflies, period of vector activity, use of traditional treatment methods, and knowledge of control measures. However, these variables did not appear as predictors of *Leishmania* infection in the final model (*p* > 0.05) (Table 5).

### 3.6. Sociodemographic Characteristics of Health Professionals

A total of 12 healthcare professionals working in Baringo county, more specifically in the *Leishmania* treatment center, were interviewed. They included 1 health record personnel, 2 medical doctors, 2 veterinarians, 2 public health officers, 2 lab technicians, 2 clinical officers, and 1 nurse, with 1–15 years of experience who were interviewed to assess their knowledge and management of leishmaniasis in Baringo County.

#### Technical Experience of Detection, Diagnoses and Treatment of *Leishmania* Infections

Participants reported that the common forms of the disease were CL, VL and post-kala-azar dermal leishmaniasis (PKDL), with 5–20 cases recorded monthly. Common clinical signs included fever, anemia, splenomegaly, hepatomegaly, skin ulcers, malnutrition, and weight loss, with children and males under 18 years being the most affected groups. Most *Leishmania* infection patients were commonly coinfected with HIV, TB, malaria, and hepatitis.

Diagnosis primarily relied on bone marrow aspiration and microscopy, supplemented by Rapid Diagnostic Tests (sensitivity 91.2%, specificity 98.3%) when available. Treatment included Sodium Stibogluconate for CL and paromomycin or liposomal amphotericin B for VL, with blood transfusions provided for severe anemia. Healthcare workers identified major challenges including shortages of diagnostic kits, drugs, blood products, trained personnel, delayed diagnosis, recurrent infections, and malnutrition. Most of the drugs and the diagnostic kits are funded by the WHO, the Kenya Medical Research Institute (KEMRI), and the Drugs for Neglected Diseases Initiative (DNDi).

While awareness and management of human leishmaniasis were generally strong, veterinarians demonstrated limited knowledge of canine leishmaniasis, highlighting a significant gap in zoonotic surveillance and control efforts in the county.

## 4. Discussion

### 4.1. Factors Associated with Knowledge, Attitude, and Practices Regarding Leishmania Infection

The present study identified a strong association between elevated awareness of leishmaniasis (94.8%) among residents of Baringo County and the region’s long-standing endemic transmission. Most respondents (86.7%) demonstrated moderate to good knowledge of the disease. This high level of awareness is further supported by reports that individuals are aware of more than 10 cases in their immediate locality. These findings align with previous reports from Baringo County, which indicate that proximity to established endemic hotspots is linked to greater disease recognition among community members [19,20,23,36] These finding are consistent with other endemic countries such as Brazil [37], Ethiopia [38], and Bangladesh [39]. Therefore, epidemiological understanding of the disease is often shaped by the visible impact of the disease within the specific social and geographic clusters of the community [20,40].

Despite high general awareness, significant knowledge gaps persist regarding the drivers of disease transmission, vector activity and ecology, and prevention. Notably, only 31.9% of respondents correctly identified sandflies as vectors of *Leishmania* infection, indicating inadequate knowledge of infection sources. Furthermore, nearly half of participants were unaware of specific breeding ecologies, such as termite mounds (anthills), and the peak activity periods of sandflies from dusk to dawn. These findings align with previous reports from Baringo County. Accurate identification of periods of high vector activity has been reported as a protective factor, indicating that temporal awareness may promote the use of preventive measures, such as bed nets, during peak sand fly biting hours [19,40,41].This lack of basic epidemiological awareness highlights a profound “knowledge–risk” gap, which limits the adoption of protective behaviors [25]. Similar knowledge gaps were reported in Central and Northwest Ethiopia [8,42], Iran [43], and Pakistan [44].

An important finding of this study is that 99.3% of respondents were unaware that domestic dogs serve as reservoir hosts for zoonotic leishmaniasis. Implications for Baringo County are significant, as pastoralist activities and close proximity to domestic animals constitute primary socio-economic risk factors in the region [19]. Although the community recognizes the disease’s clinical signs in humans, there is limited understanding regarding the role of domestic dogs in the zoonotic mechanisms due to the lack of visible clinical signs, which may foster a false sense of security among owners [45,46]. This lack of awareness about canine reservoirs aligns with findings from other East African pastoral communities, including those in Ethiopia [8] and other parts of Kenya, where the animal–human transmission interface is often overlooked in public health education [19]. In contrast, in regions where canine leishmaniasis surveillance is more established, such as Oromia, Ethiopia [27,47], and Brazil [37], studies have demonstrated high seroprevalence rates in dog populations, emphasizing the potential risk to human health. Therefore, increased community awareness of dogs’ role in the transmission cycle in endemic regions is essential, as knowledge gaps pose a significant challenge for One Health-based control strategies. To reduce the disease burden in Baringo County, there is an urgent need for One Health education programs that integrate human and animal health perspectives, with a particular focus on enhancing community understanding of the zoonotic reservoir system [27,48].

The study identified substantial knowledge gaps regarding vector prevention and control measures. Most respondents were unaware of environmental control practices, including vegetation clearance and destruction of termite hills. Comparable deficiencies have been reported in Ethiopia [8], Sri Lanka [10], and Pakistan [44]. Such gaps may impede the adoption of effective preventive strategies and perpetuate disease transmission within the community.

Overall, respondents demonstrated a positive attitude (76%) towards leishmaniasis with 82.2% of respondents demonstrating positive attitude toward treatment through willingness to seek medical care at healthcare facilities when they feel sick. This is significant because a negative attitude was statistically associated with delays in early detection and diagnosis, which increases the risk of persistent infection. Such delays, often caused by logistical barriers and the remoteness of pastoralist settlements, contribute to higher morbidity and mortality in endemic areas like Baringo [21,24,30]. This level of health-seeking intent is consistent with other endemic regions in East Africa where community awareness of the disease’s severity is high. Comparable findings have been reported in previous studies in Baringo [19] and Ethiopia [28,31].

A notable contradiction emerges as 68.9% of respondents perceive hospital treatment as prohibitively expensive. This perception constitutes a significant socio-economic barrier to accessing healthcare in facilities such as Kimalel or Chemolingot in the remote sub-counties of Baringo South and Tiaty and may contribute to 29.6% of participants opting for traditional remedies. Although this association did not reach statistical significance, the findings indicate that cultural beliefs regarding disease management are prevalent among both infected and non-infected households in these endemic areas. Consequently, modern healthcare is frequently deferred due to physical barriers, extended travel distances, and security risks associated with accessing medical facilities [19,21,24]. While some residents noted that treatment is available for free through specialized programs (such as DNDi at the Kimalel Health Center), the widespread fear of high costs suggests a need for better communication regarding the availability of subsidized or free care to prevent delays in diagnosis [29,36]. Comparable findings were reported in similar studies elsewhere in Ethiopia [38] and Sri Lanka [10].

The study highlights that leishmaniasis is perceived as a primary public health threat in Baringo, with 82.2% of residents finding it a major community burden. Notably, many participants perceive the disease as a greater problem than malaria [19,49]. This perception likely stems from the chronic nature of the disease, the social stigma associated with splenomegaly or cutaneous scars, and the perceived lack of resources compared to the highly visible malaria control programs in the region [30]. Similar reports on leishmaniasis being perceived as a community have been reported in Sodo Ethiopia [38] and Pakistan [44].

However, only age and educational attainment emerged as significant predictors of attitudes towards the disease. These findings indicate that older community members may have developed more serious perceptions of leishmaniasis due to prolonged exposure and observation of the clinical impact of previous outbreaks in Baringo [19]. Furthermore, higher education levels serve as a significant protective factor, likely attributable to increased literacy and improved access to health information [25]. These results are consistent with studies from other endemic regions, where educational attainment is identified as a key determinant of community perceptions and responses to neglected tropical diseases [20,25,47,50]. Therefore, targeted health education programs should specifically focus on younger populations and those with no formal schooling to bridge the identified attitudinal and behavioral gaps [19].

Preventive practices among respondents were generally favorable (77.7%), particularly in the use of bed nets and environmental sanitation through proper garbage disposal. Notably, 96.3% of respondents reported sleeping under protective bed nets, a result attributed to established malaria control initiatives in the Baringo region. Although these nets are primarily intended to target Anopheles mosquitoes, they also serve as a secondary barrier against sand flies, which are nocturnally active. Integrating leishmaniasis prevention into existing malaria distribution and education programs may offer a cost-effective strategy with additional benefits for reducing transmission in these pastoralist communities [19,41,49]. However, there was no association between high net usage and infection status, possibly due to the outdoor biting nature of certain sand fly species in the region [40].

Preventive measures such as clearing vegetation and removing termite hills can significantly reduce vector density. Termite hills continue to pose an environmental challenge, highlighting the importance of environmental sanitation and targeted vector control. This observation is consistent with evidence from Baringo South and Tiaty, where termite hills were identified as primary breeding and resting sites for the phlebotomine sand fly vector [19,20]. However, these associations were not statistically significant, due to the homogeneity of practices within the pastoralist community and the logistical constraints of the 135-respondent sample size [19,41]. 

Another significant finding regarding preventive measures was that 97.2% of the residents practiced proper garbage disposal. This proper garbage disposal, on the other hand, reduces sandfly breeding sites for sandfly vectors. In addition, garbage accumulation could attract rodents, increasing the risk of infection. This aligns with similar studies in Pakistan [51] and Iran [52,53]. This suggests that while residents are willing to engage in environmental management to reduce vector density, there is lower motivation for consistent personal protection, due to the logistical or cultural challenges of supporting these habits in a pastoralist setting [40].

Although preventive practices are in place, significant behavioral risks persist, as 24.4% of individuals report sleeping near their animals at night. Close contact between humans and animals remains a critical risk factor for zoonoses in this region, where 93.3% of the population rear domestic animals such as cattle, sheep, and goats, and 81.5% own dogs. Previous studies in Baringo have documented these practices and highlighted a lack of awareness regarding the role of dogs as reservoirs. Such behaviors may facilitate host–vector interactions in areas with high sand fly activity, thereby increasing the risk of exposure to zoonotic leishmaniasis (*Leishmania infantum*) [19,27,40,41]. Moreover, in pastoralist settings like Baringo, the presence of livestock near dwellings can increase vector density by providing alternative blood meals for sand flies. In addition, animal proximity is a significant risk and behavioral changes often only occur after an initial infection [19,40]. Similar studies elsewhere in Western Tigray and Oromia Ethiopia [31,47], Pakistan [51], Bangladesh [7], and Iran [53] have also reported similar practices that increased the risk of *Leishmania* infection in human.

The study found that seasonal migration linked to pastoralism, farming, and trade can contribute to the spread of infection and hinder disease control. Temporary settlements in highly endemic or termite-hill-dense areas increase exposure to sand fly vectors and make consistent access to healthcare and follow-up more difficult [19,21]. Population movement has previously been associated with expansion of leishmaniasis transmission into new endemic areas [54].

Although these risks were identified, the study did not find a statistically significant association between specific preventive practice variables and infection status. This outcome suggests that pervasive environmental risk in Baringo South and Tiaty may have a greater influence than individual behavioral interventions. Furthermore, the lack of statistical significance suggests that preventive knowledge is relatively uniform across the population, likely due to prolonged exposure to the disease [19].

### 4.2. Capacity of the Healthcare System

The present study demonstrates that healthcare workers possess adequate knowledge regarding the diagnosis and management of leishmaniasis. Additionally, subsequent reports indicate that *Leishmania* infection disproportionately affects males and individuals under 18 years of age. This demographic vulnerability may be attributed to pastoralist activities and the tendency to remain outdoors late at night, which increases exposure to sand fly vectors [30]. Moreover, the study identifies a significant association between leishmaniasis and immunosuppressive co-infections such as HIV, tuberculosis, and malaria, suggesting that immunocompromised individuals in Baringo are at a substantially higher risk of developing severe disease [30]. Similar findings have been documented in other endemic regions in Africa [31,55] and in Portugal [56].

Healthcare workers demonstrate strong technical skills in managing the disease amid recurrent outbreaks. However, the healthcare system faces major operational challenges, including shortages of diagnostic kits, limited access to essential drugs, and insufficient blood products for transfusions. Bone marrow aspiration for amastigote identification remains the primary diagnostic method, as more sensitive tools such as RDTs are often unavailable due to funding constraints [30]. Similar operational challenges have been documented in other endemic regions in Africa [31,55].

Furthermore, the study proved that veterinarians in Baringo lacked sufficient knowledge about canine leishmaniasis detection, diagnosis, and treatment. This highlights significant disparity in the knowledge gaps and critical oversight in recognition of the zoonotic dimension of the disease [27,48,57]. The lack of awareness among veterinary professionals about the risk posed by domestic dogs is a major barrier to integrated disease control and underscores the need for a One Health approach to surveillance. The small number of veterinary participants reflects the scarcity of veterinary resources dedicated to zoonotic surveillance in Baringo. Their input is critical for identifying the “One Health” knowledge gap and the disconnect between human and animal health surveillance which was a primary focus of this research.

### 4.3. Study Limitations

Its cross-sectional design prevents establishing causal relationships between identified risk factors and leishmaniasis infection. The relatively small sample size and inclusion of only one ethnic group (Kalenjin) limit the generalizability of the findings. Reliance on self-reported data may have introduced recall and social desirability biases, and participants’ knowledge and perceptions may not accurately reflect actual practices. The findings are also specific to the semi-arid environment of Baringo South and may not be applicable to other endemic regions.

In addition, limited diagnostic capacity and the absence of comprehensive assessments of canine leishmaniasis and vector density may have affected the accuracy and completeness of understanding local transmission dynamics.

## 5. Conclusions

The study finds that although human leishmaniasis is widely recognized as a major community health issue, its control in Baringo County, Kenya, is hindered by significant knowledge and operational gaps. While general awareness is high, there are persistent gaps in understanding disease transmission, vector ecology, prevention, and the zoonotic role of dogs. High-risk behaviors, including residents sleeping near dogs, further increase host–vector–human contact.

The local healthcare system is technically capable but constrained by ongoing shortages of diagnostic kits and medications, resulting in delayed diagnoses and higher morbidity. The study recommends adopting a One Health approach that integrates human and veterinary surveillance. Effective control in Baringo requires bridging the gap between human health management and the overlooked canine reservoir, as well as utilizing existing malaria control resources to encourage bed net use.

## Figures and Tables

**Table 1:** Association of sociodemographic characteristics and KAP of the respondents in Baringo County, Kenya (*n* = 135).

Variable	Category	Frequency/%	*p* Value		
			Knowledge	Attitude	Practice
Gender	Male	75 (55.6)	0.302	0.273	0.703
	Female	60 (44.4)			
Age (years)	Mean ± SD	42.6 ± 14.6	0.635	0.07	0.668
	Range	18–83			
Resident	Yes	131 (97%)	0.423	0.034	0.803
	No	4 (3)			
Education level	None	13 (9.6)	0.180	0.014	0.617
	Primary school	59 (43.7)			
	Secondary school	41 (30.4)			
	Tertiary	22 (16.3)			
Family role	Father	65 (48.1)	0.553	0.373	0.382
	Mother	58 (43)			
	Children	12 (8.9)			
Marital status	Married	100 (74.1)	0.762	0.001	0.069
	Single	19 (14.1)			
	Widowed	15 (11.1)			
	Divorced	1 (0.7)			
Occupation	Mixed farming	76 (56.3)	0.381	0.016	0.650
	Casual laborer	25 (18.5)			
	Trading	21 (15.6)			
	Other employment	13 (9.6)			
Ever diagnosed	No	113 (83.7)	---	---	---
	Yes	22 (16.3)			

Note: SD = standard deviation.

**Table 2:** Results of univariate analysis on knowledge indicators related to leishmaniasis among residents in Baringo County, Kenya (*n* = 135).

Knowledge Indicators	Category	No	%	*p* Value
Have heard of Leishmaniasis	Yes	128	94.8	0.231
	No	7	5.2	
Awareness of reported cases in the locality	No	51	37.8	0.011
	Yes	84	62.2	
Awareness of at least one clinical sign	Yes	135	100	0.007
	No	0	0	
Source of disease information	Local community	57	42.2	0.227
	Healthcare workers	40	29.6	
	Media (radio/TV)	21	15.6	
	Other media sources	17	12.6	
Knowledge of sources of infection	Sandflies	43	31.9	0.078
	Domestic animals	4	20.7	
	Do not know	64	47.4	
	Termite hills	24	17.8	
Awareness of native name of the disease	Kala azar	17	12.6	0.012
	Other names	54	40	
	No	64	47.4	
Awareness of presence of biting flies	No	24	17.8	0.017
	Yes	111	82.2	
Knowledge of vector activity	Morning	83	61.5	0.055
	Evening	28	20.7	
	Unknown	24	17.8	
Awareness of dogs as reservoir hosts	Yes	1	0.7	0.519
	No	134	99.3	
Awareness of preventive measures	Yes	54	40	0.686
	No	81	60	
Awareness of control measures	Yes	65	48.1	0.036
	No	70	51.9	

**Table 3:** Results of univariate analysis of attitudes indicators related to leishmaniasis among the respondents.

Attitude Indicators	Response	*n*	%	*p* Value
Seek medical treatment if sick	Yes	111	82.2	0.076
	No	24	17.8	
Leishmaniasis as community problem	Yes	111	82.2	0.076
	No	24	17.8	
Preferred treatment method	Hospital treatment	93	68.9	0.040
	Traditional remedies	18	13.3	
	Not sure	24	17.8	
Perception of the treatment cost	Expensive	93	68.9	0.040
	Affordable	18	13.3	
	Not sure	24	17.8	
Individual prevention measures	Agree	54	40	0.686
	Disagree	81	60	
Community control measures	Agree	65	48	0.036
	Disagree	70	51.9	

**Table 4:** Results of univariate analysis of preventive practices indicators among the respondents in Baringo County.

Practice Variable	Category	*n*	%	*p* Value
Sleeping behavior	Inside the house	129	95.6	0.980
	Outside the house	6	4.4	
Use of protective net	Yes	130	96.3	0.819
	No	5	3.7	
Dog ownership	Yes	110	81.5	0.519
	No	25	18.5	
Sleeping near dogs	Yes	33	24.4	0.455
	No	102	75.6	
Use of traditional medicine	Yes	40	29.6	0.205
	No	95	70.4	
Waste disposal method	Burning	129	95.6	0.543
	Burial	3	2.2	
	None	3	2.2	
Migration	No	59	43.7	0.115
	Yes	76	56.3	

**Table 5:** Results of logistic regression analysis of factors associated with awareness and attitude of the respondents regarding leishmaniasis.

Variable	Category	Odds Ratio (OR)	95% CI	*p*-Value
Awareness of reported cases	Yes	5.76	0.95–43.69	0.058
No of cases	>10	1.752	0.203–15.625	0.613
Knowledge of infection source	Don’t know	7.78	1.42–52.87	0.018
Knowledge of infection source	Termite hill and sandflies	4.42	0.77–31.36	0.097
Treatment method	None	11.51	0.30–495.64	0.185
	Traditional	1.506	0.327–6.949	0.599
Vector activity awareness	Morning	0.82	0.18–3.35	0.779
Knowledge of control measures	Clear vegetation	0.849	0.073–9.468	0.895
	Control flies	0.522	0.036–7.53	0.633
	Destroy termite hills	0.474	0.047–4.709	0.525
	None	0.217	0.020–2.334	0.208

## Data Availability

Data supporting the findings of this study are available from the corresponding author upon reasonable request.

## Abbreviations

The following abbreviations are used in this manuscript:

VL	Visceral Leishmaniasis
CL	Cutaneous Leishmaniasis
KAP	Knowledge, Attitudes and Practices
WHO	World Health Organization
PKDL	Post-Kalzar-Dermal Leishmaniasis
MCL	Mucocutaneous Leishmaniasis
NTD	Neglected Tropical Diseases
SSG	Sodium Stibogluconate
HIV	Human Immunodeficiency Virus
TB	Tuberculosis
RDT	Rapid Diagnostic Test
AIDS	Acquired Immunodeficiency Syndrome
DNDI	Drugs for Neglected Diseases Initiative
KEMRI	Kenya Medical Research Institute
KNBS	Kenya National Bureau of Statistics
